# Welfare Effects of the Use of a Combination of Local Anesthesia and NSAID for Disbudding Analgesia in Dairy Calves—Reviewed Across Different Welfare Concerns

**DOI:** 10.3389/fvets.2018.00117

**Published:** 2018-06-05

**Authors:** Mette S. Herskin, Bodil H. Nielsen

**Affiliations:** Department of Animal Science, Aarhus University, Tjele, Denmark

**Keywords:** pain, calf, NSAID, affective state, dehorning, welfare, behavior

## Abstract

Across the international dairy industry, the vast majority of dairy herds have dehorned cows. Farmers choose to dehorn calves for different reasons such as easier handling of non-horned cattle and reduced risk of injuries in animals and staff. This review focuses on disbudding by use of hot-iron cautery as is practiced by dairy farmers in calves <3 months of age. We examine welfare effects of the use of NSAIDs in combination with a local anesthetic including indicators relevant for the three major welfare concerns—affective states, basic health, and functioning as well as the possibility to behave naturally. Across indicators of animal welfare, the majority of available evidence suggest that the use of NSAIDs in combination with a local anesthetic is advantageous in terms of animal welfare, and no studies suggest that NSAIDs are a disadvantage to animal welfare. However, irrespective of the welfare concern, use of NSAIDs combined with a local anesthetic does not fully eliminate the welfare challenges from disbudding. Further research is needed in order to fully understand the effects of this medication protocol on calf welfare, including knowledge about the duration of healing and the presence of long-term pain. At present, this lack of knowledge challenges the precise formulation of adequate pain management—in terms of medication protocol, duration, dosage, and type of administration.

## Introduction

Even though breeding for polledness is receiving increased focus [e.g., (1)], the vast majority of dairy herds have dehorned cows [e.g., (2)]. Farmers choose to remove horns from calves for different reasons. Making the handling of cattle easier and reducing the risk of injuries (in cattle, other animals and humans) inflicted by horned cattle are stated as primary reasons for removal of horns or horn buds (2). In addition, financial (animals without horns are easier sold and at a higher price) as well as aesthetic reasons have been put forward (3).

Disbudding refers to removing or destructing the horn bud and the horn producing cells before it is attached to the underlying tissue—typically involving calves younger than 3 months—whereas dehorning refers to removing the horn after its attachment to the underlying tissue (4). This review focuses on hot-iron disbudding. In Denmark, this is currently the only legal disbudding procedure and it is obligatory that a veterinarian applies a local anesthetic before the procedure (5). Optionally, calves can be sedated in order to minimize the stress of the calves during the procedure and ease the work. Some veterinarians choose to supplement with non-steroidal anti-inflammatory analgesics [NSAIDs, inhibiting synthesis of inflammatory mediators such as prostaglandins and leukotrienes (6, 7)] as part of the procedure.

Across countries and regions, farmers as well as veterinarians consider removal of horn buds a painful procedure for calves (8–11). Irrespective of the applied technique, the many available studies come to the same conclusions—the procedure leads to severe pain [as reviewed by (12, 13)], and has negative consequences for the welfare of the calves. However, understanding of animal welfare is influenced by value-based concerns about requirements for a good animal life (14, 15) which can be grouped under three headings: (a) basic animal health and production; (b) the affective states of animals; and (c) the ability of animals to live naturally.

This paper reviews welfare effects of the use of a combination of local anesthesia and NSAID as pain relief during and after hot-iron disbudding in dairy calves. The assessment of the welfare effects includes all three major welfare concerns. This critical review (16) is based on relevant scientific literature from the databases Web of Science and Google Scholar collected in October 2017 with the following key words “dehorning” and “disbudding” and limited to studies involving cattle.

## Animal welfare: definition and indicators

Animal welfare is a multifactorial, international and domestic public-policy subject, incorporating scientific, ethical, and economic issues as well as religious, cultural, and trade considerations (17). Scientifically, animal welfare can be referred to as the state of an animal and how it copes with its environment (18). Animal welfare can be studied scientifically, but is not directly measurable and must be assessed by use of indicators. In this review, we organize indicators of animal welfare in five, not completely mutually exclusive categories used for overview: behavioral indicators, clinical indicators, blood parameters, production parameters, and indicators of the affective states of the animal. Across the categories, we include measures of basic health and functioning, of affective states, and the possibility to behave naturally, thereby following recommendations from Fraser (15) to include aspects of all three typical welfare concerns.

Traditionally, measures of health and functioning of farm animals have been central to the evaluation of animal welfare, based on the need to maintain production and avoid diseases (19). More recently, the importance of measures of animal affective states, such as pain and fear, as part of welfare assessment, have been debated in the scientific literature [e.g., (20, 21)]. Today, evidence for affective states (negative as well as positive) is one of the major animal welfare concerns. The evaluation of such states is now included in welfare assessment schemes such as Welfare Quality® (22), where the positive emotional state of calves is assessed by observation of play behavior. What exactly constitutes naturalness is open to interpretation (23), but according to Ventura et al. (24), concerns seem to be expressed in terms of the physical environment in which animals are kept, with priority given to expression of natural behavior and freedom of movement, and often at least some outdoor access.

Despite their distinct characteristics, the three major welfare concerns suggested by Fraser et al. (14) share many indicators of animal welfare. Strict grouping of the available indicators according to welfare concern is complicated and lies beyond the scope of this review. One example, drawn from the available studies of hot-iron disbudding, is observations of play behavior (25). Play behavior is considered important for the affective state of the calves [as an indicator of positive affective state (26)], but also relevant for the concern of the naturalness of the calves as an expression of natural behavior. Table 1 gives a schematic overview of the categorization of the welfare indicators used in this review and their relevance for the three welfare concerns. As indicators of affective state and behavior overlap to a large degree, these are shown together but marked differently.

**Table 1:** Schematic overview of the categorization of the five (not mutually exclusive) categories of welfare indicators mentioned in this review as well as their relevance for the three major welfare concerns: health and functioning, affective state, and natural living (14).

**Indicator of calf welfare**	**Health and functioning**	**Affective state**	**Natural living**
**BEHAVIORAL INDICATORS**			
Feeding	X	X	X
Drinking	X	X	X
Lying	X	X	X
Standing	X		
Lying inert	X	X	
Locomotion	X		X
Grooming	X	X	X
Self-grooming	X	X	X
Exploring		X	X
Postural changes		X	X
Play behavior^b^		X	X
Rumination^b^	X	X	
Head shake^b^		X	
Head rub^b^		X	
Ear flick^b^		X	
Head-out-of-pen^b^		X	
Tail flick^b^		X	
Foot stamp^b^		X	
Falling^b^		X	
Escape attempt^b^		X	
Rearing^b^		X	
Struggling^b^		X	
**CLINICAL INDICATORS**			
Respiration	X	X	
Heart rate/heart rate variability	X	X	
Ocular temperature	X		
Nociceptive threshold^a^	X		
Horns or horn buds removed			X
Degree of healing	X	X	
Bleeding	X		
**BLOOD PARAMETERS**			
Plasma cortisol	X	X	
Haptoglobin	X	X	
Prostaglandins	X	X	
Circulating neutrophils	X		
Substance P	X	X	
**PRODUCTION INDICATORS**			
Changes in body weight	X		X
Feed intake/milk consumption	X		X
**AFFECTIVE STATE INDICATORS**			
Behavioral indicators marked by^b^		X	
Human verbal report of pain score		X	
Pain scale or pain score		X	
Cognitive bias		X	
Conditioned place avoidance		X	

*Importantly, strict grouping of the indicators according to welfare concern is complicated and lies beyond the scope of this review. Hence, the distinctions made are not conclusive and reflects decisions made by the authors in order to create overview and stimulate discussion. As indicators of affective state and behavior overlap to a large degree, these are shown together and indicators often interpreted in relation top affective state are marked*.

a *Changes in nociceptive threshold are a well-described consequence of tissue damage, inflammation and pain but not a measure of affective state as such*.

b *Behavioral indicators often interpreted in relation to affective state*.

## Disbudding and animal welfare—mainly focusing on pain

Removal of horns is among the most examined tissue damaging management procedures in cattle—several studies have been conducted focusing on different types of dehorning and disbudding, such as amputation (27–31), chemical disbudding by caustic paste (32) and hot-iron disbudding. Until now, the vast majority of studies have focused on the consequences of the management procedure in terms of pain or stress. Concurrently, it is agreed that hot-iron disbudding without any analgesics is severely painful for calves, as evidenced by for instance (33–43) and as reviewed by Hambleton and Gibson (3), Stafford and Mellor (13), and Stock et al. (44). Much less agreement can be found regarding the duration of the pain after removal of horns in calves. Some studies suggest that the pain is present for only a few hours (45, 32), up to 24 h (46), or potentially up to 44 h (where their data collection was terminated) (38).

The pain experienced at disbudding is comprised of at least three pain modalities: (1) acute nociceptive pain in response to tissue damage; and (2) inflammatory pain which may persist for days or weeks until the tissue damage is resolved; and (3) neuropathic pain, which occurs when the somatosensory nervous system itself is damaged [as recently reviewed by (47) for bovine pain]. In cases like this, effective pain management needs to be multimodal and target more than one underlying mechanism to cover all pain involved. However, the term “multimodal analgesia” may not always be concisely defined. For example, the term is used to describe any combination of analgesic drugs acting on different pathways (48) and to describe the specific combination of opioids and regional blocks (attenuation of pain-related signals in the central nervous system) and NSAIDs (acting mainly in the periphery to inhibit the initiation of pain) (49). Recently Winder et al. (50) did a systematic review and meta-analysis of effects of local anesthetic or systemic analgesia on selected measures of pain after hot-iron disbudding in calves. In this critical review, we focus on the welfare effects of NSAID in combination with local anesthesia compared to unimodal local anesthesia alone.

Local anesthetics have proved effective in handling the acute nociceptive pain during the procedure of horn removal (37, 51, 52). Local anesthesia can be achieved by a cornual nerve block. Typically, 5 ml of 2% Lidocaine- or Procainehydrochloride are injected around the cornual nerves located under the temporal ridge at each side of the face of the calf. The cornual block can be supplemented with subcutaneous infiltration of a local anesthetic around the horn basis (ring block) (52, 53). Fierheller et al. (54) tested the efficacy, the onset and the duration of cornual nerve block and ring block with Lidocaine 2% in calves. Both methods had a rapid onset within few minutes and lasted approximately 5 h and two and a half hour, respectively. Other studies of disbudding have reported that the local anesthetic action wears off within 2–3 h, which is supported by the review of physiological, clinical as well as behavioral pain indicators below.

Following the fading of the local anesthetic effect, pain arises primarily caused by products of the inflammatory processes initiated by the tissue damage, suggesting that longer lasting pain relief is needed in order to diminish the negative welfare consequences of horn removal. During the last two decades, the effects of administration of NSAIDs at horn removal have been investigated. The rationale for using NSAIDs lies in the nature of their effects as they block the production of inflammatory mediators such as prostaglandins (55). The effects of NSAIDs combined with local anesthetics compared to local anesthetics alone as pharmacological relief of post-disbudding pain are relatively well-documented [and recently presented in a systematic review (50)]. Less is known about the effects of NSAIDs in a broader welfare perspective. This is reviewed below and for overview, the different findings are categorized into the five different types of welfare indicators introduced above.

## Consequences of disbudding for calf welfare—effects of pain relief

### Behavioral indicators

The behavior of farm animals, including calves, is considered a key indicator of their welfare [e.g., (56, 57)] and has been studied considerably in relation to tissue damaging management routines such as removal of horns [as reviewed by for example (12)]. The majority of the available studies involving behavioral data have focused on the immediate response behaviors such as vocalizations, kicking, and falling; [e.g., (51)] as indicators of pain—these are presented in section Indicators of Affective States considering indicators of affective state and indicated as such in Table 1.

Few studies have included quantification of the occurrence or duration of non-evoked behavioral states or events such as locomotion, self-grooming, or drinking (38, 46, 58, 59). In a study comparing post-disbudding behavior of calves administered NSAID compared to non-treated controls, Theurer et al. (60) found increased lying for 1–4 days after disbudding. A few other studies of time budgets involved recordings of lying behavior (39, 43). In one of the early studies, Grøndahl-Nielse et al. (35) quantified the latency to resume rumination after hot-iron disbudding and Sylvester et al. (31) recorded the occurrence of rumination in their study of behavior after amputation dehorning.

The only available paper, studying the behavior of NSAID-treated calves after disbudding in a standardized behavioral test or motivational paradigm is Mintline et al. (25), showing increased occurrence of play in NSAID-treated calves when the animals were tested 3 h post-disbudding.)(Table 2). Play behavior in calves can be considered important for the welfare concern of natural living. However, it is also considered as an indicator of positive affective state (26) and is also included in the calf version of the welfare assessment protocols of Welfare Quality® (22). As discussed by Mintline et al. (25) examination of the relation between removal of horns and play is a practical model to test the systemic effect of the tissue damaging procedure, as the resulting discomfort or tissue damage are unlikely to directly affect locomotion—the calves may not play, but they are able to do it. Rushen and De Pasillé (67) found reduced play in calves on day 4 after chemical disbudding.

**Table 2:** List of publications (by year of publication) involving hot-iron disbudding of calves and the comparison of calves disbudded after administration of a local anesthetic (LA) vs. LA + NSAID.

**References**	**Age range and number of calves per treatment**	**Analgesics used and interval to disbudding (sedation not mentioned in this table)**	**Variables involved**	**Effects of combination of NSAID and LA when compared to LA**
(46)	4–8 weeks, *N* = 10	Lidocaine (10 min) Ketoprofen (−2, 2, 7 h)	Head shake (3 to 24 h) Ear flick (3 to 24 h) Head rub (3 to 24 h) Feeding (3 to 24 h) Exploring (3 to 24 h) Locomotion (3 to 24 h) Self–grooming (3 to 24 h) Head–out–of–pen (3 to 24 h) Vocalization (3 to 24 h) Lying (3 to 24 h) ADG^a^ (24 h)	↓3–12 h ↓3–24 h ↓4–12 h No effect No effect No effect No effect No effect No effect No effect Tended to ↑
(58)	2–14 days, *N* = 20	Lidocaine (10 min) Ketoprofen (10 min)	Cortisol (0 to 6 h) Ear flick (0 to 8 h) Head shake (0 to 8 h) Head rub (0 to 8 h) Lying (0 to 8 h) Feeding (0 to 8 h) Drinking (0 to 8 h) Self–grooming (0 to 8 h) Feed intake (24 h)	↓change from 0 to 3 h No effect No effect No effect No effect No effect No effect No effect No effect
(61)	6–12 weeks, *N* = 30	Lidocaine (10 min) Meloxicam (timing not reported)	Cortisol (0 to 24 h) Heart rate (0 to 24 h) Respiration (0 to 24 h)	↓0–6 h ↓overall ↓0–6 h
(52)	4–5.5 weeks, *N* = 8	Lignocaine hydrochloride (10 min) Meloxicam (55 min)	Heart rate (0–180 min) Heart rate variability (0–180 min) Ocular temperature (0–180 min)	↓increase when LA wanes off Differed from LA No clear effects
(59)	4–8 weeks, *N* = 20	Lidocaine (10 min) Ketoprofen (10 min)	Cortisol (3 to 6 h) Ear flick (0 to 8 h) Head shake (0 to 8 h) Head rub (0 to 8 h) Total head behaviors (24 h) Lying (0 to 8 h) Standing (0 to 8 h) Feeding (0 to 8 h) Grooming (0 to 8 h) Feed intake (24 h)	No effect ↓overall No effect No effect ↓overall No effect No effect No effect No effect Tended to ↑
(38)	6–12 weeks, *N* = 30	Lidocaine (10 min) Meloxicam (10 min)	MNT^b^ (4 h) Ear flick (−22 to 44 h) Head shake (−22 to 44 h) Head rub (−22 to 44 h) Tail flick (−22 to 44 h) Foot stamp (−22 to 44 h) Head through bars (−22 to 44 h) Feed intake (24 h) Activity (48 h)	↑threshold ↓overall ↓4–9 h No effect No effect No effect No effect Tended to ↑ ↓ 0–5 h
(40)	8–10 weeks, *N* = 6–7	Lidocaine (15 min) Carprofen (15 min)	Cortisol (0 to 24 h) Inert lying (0 to 24 h) Head shaking (0 to 24 h) Ear flicking (0 to 24 h) Head rubbing (0 to 24 h) Behavioral transitions (0 to 24 h) Sum of behaviors (0 to 24 h)	Comparable to sham except for 24 h Not observed ↓at 15 min ↓at 3 and 6 h ↓at 3 h No effects No effects
(41)	8–10 weeks, *N* = 10	Lidocaine (10 min) Meloxicam (0 or 12 h)	Cortisol (0–7 d) Substance P (0–7 d) Haptoglobin (0–7 d) PGE_2_^c^ (0 to 72 h)	↓increase when LA wanes off ↓at 120 h No effect ↓up to 48 h
			MNT^b^ (0–7 d) ADG^a^ (7 d) Ocular temperature (0 to 12 h)	↓threshold at 3 h No effect No effect
(42)	5–9 weeks, *N* = 20	Procaine (20 min) Flunixin (20 min, 3t)	Cortisol (−1.25 to 8 h) Heart rate (−1.25 to 8 h) Respiration (−1.25 to 8 h) Head shaking (−1.25 to 8 h) Head rubbing (−1.25 to 8 h) Foot stamping (−1.25 to 8 h) Ear flicking (−1.25 to 8 h) Groaning/moaning (−1.25 to 8 h) Head protrusion (−1.25 to 8 h)	↓AUC^d^ No effect No effect No effect No effect No effect No effect No effect No effect
(25)	4–5.5 weeks, *N* = 8	Lignocaine hydrochloride (10 min) Meloxicam (55 min)	Play behavior at 3 and 27 h Von Frey filaments −1 to 75 h	↑play at 3 h Thresholds not affected
(62)	3–6 weeks, *N* = 51	Lignocaine hydrochloride (0 min) Meloxicam (0 min)	ADG^a^ (15 d, 30 d) Milk consumption (0–11 d)	No effect No effect
(63)	<2 mon, *N* = 8–10	Adrenacaine (10–15 min) Meloxicam (10–15 min)	Heart rate variability (48 h)	No effect
(64)	4–6 weeks, *N* = 10	Lidocaine (10 min) Firocoxib (10 min)	Cortisol (0 to 96 h) Substance P (0 to 96 h) Heart rate (0 to 24 h) MNT^b^(0 to 24 h) Ocular temperature (24 h) ADG^a^ (7 d) PGE_2_^c^ (72 h)	↓AUC^d^ No effect No effect No effect No effect No effect ↓ up to 48 h
(65)	3–6 weeks, *N* = 29–73	Lignocaine hydrochloride (0 min) Meloxicam (0 min)	ADG^a^ (15 d, 30 d)	No effect
(66)	51 ± 5 d, *N* = 10	Lidocaine (5 min) Carprofen (5 min)	Cortisol (96 h) Substance P (96 h) MNT^b^ (96 h) Ocular temperature (96 h) Heart rate (96 h) ADG^a^ (7 d) PGE_2_^c^ (96 h)	No effect No effect No effect No effect No effect No effect ↓ overall

*Experiments using other types of horn removal or not involving this specific comparison (e.g., when only one of the groups has been sedated) have been left out of the table*.

a *ADG, average daily gain*.

b *MNT, mechanical nociceptive threshold obtained with hand held algometer*.

c *PGE_2_, plasma concentration of prostaglandins*.

d *AUC, area under the curve*.

### Clinical indicators

In this review, the category of clinical indicators includes measures that can be either directly observed on the calves or recorded with non-invasive devices such as heart rate monitors or thermography.

Irrespective of possible pain management, removal of horns breaches the integrity of the animals. Hot-iron disbudding destroys a ring of skin causing 3rd degree burns where the hot iron is applied and 1st and 2nd degree burns on surrounding tissue [as reviewed by (47)]. Only very few studies have sought to quantify the clinical changes induced by procedures of horn removal or to quantify healing of the induced lesions. Huebner et al. (68) started systematic focus on the healing of the lesions induced by hot-iron disbudding. AVMA (69) described, without direct evidence, that hot-iron disbudding is advantageous as it leads to less bleeding and fewer complications during healing than removal of horns by other procedures such as chemical or surgical disbudding. Huebner et al. (68) scored lesions (scale from 1 to 3) and wound diameter during 3 weeks post-disbudding. All calves in the study still scored 1 in the third week, hence the lesions were not healed. Wound diameter was 16 mm in week 1 and only reduced to 15.5 mm in week 3.

Quantification of mechanical or thermal nociceptive threshold measures of the animals' sensitivity toward nociceptive stimuli of different sensory modalities and is, thus, not a measure of affective states as such. Hence, with the present categorization of indicators, we have chosen to discuss changes in nociceptive threshold as a clinical indicator. Changes in sensitivity are a well-described consequence of tissue damage, inflammation, and pain (70). The finding of a lower nociceptive threshold in injured animals compared to intact control animals, suggests that the stimulated skin area has an increased sensitivity (hyperalgesia). This can potentially be explained by inflammation-induced changes in the sensitivity of local nociceptors or by central changes (71), and has been shown in human burn patients in response to mechanical as well as thermal stimulation (71, 72). Both primary (at the injured tissue, mediated by peripheral nociceptors, immediate) and secondary (at surrounding tissue, mediated by spinal neurons) hyperalgesia may occur immediately after injury, but the secondary hyperalgesia may take hours before reaching its peak. The ability to recruit otherwise silent nociceptors may play a role in primary hyperalgesia after burn injury (71). Increased sensitivity of the injured as well as surrounding skin have been reported from horn removal studies in calves, typically quantified as mechanical nociceptive threshold when stimulated by an algometer (MNT) (41, 64) or by use of von Frey filaments (25, 73, 74).

Also studies examining effects of NSAID on post-disbudding responses of calves have involved quantification of mechanical nociceptive thresholds by algometers (38, 41, 64, 66) or by von Frey filaments (25), however, covering rather variable time periods after the tissue damaging management procedure. Only (38) and (41) found effects of NSAIDs and in opposite directions (Table 2). Recently, (75) reported increased MNT and increased sensitivity toward von Frey filament stimulation in calves after disbudding. However, in their study, all calves were administered preventive multimodal analgesia, and no untreated control calves were involved. Thus the isolated effects of NSAID cannot be inferred from this study.

Other clinical measures recorded in studies of the immediate response of calves to disbudding are respiration [which has been shown to increase (76)], and other physiological indicators of sympathetic activation. One such indicator is heart rate (37, 39, 52, 76). Grøndahl-Nielsen et al. (35) found increased heart rate for 3.5 h after hot-iron disbudding in calves receiving no pain relief. More recent studies have included measures of heart rate variability, suggested to reflect autonomous activation via assessment of sympathetic and parasympathetic activation, and therefore possibly a measure of pain (77). However, even though some studies have reported effects of the use of local anesthetics on these measures, the results have been diverging and difficult to interpret. Further studies are needed in order to understand the link between hot-iron disbudding in calves and their heart rate variability.

Indicators of sympathetic activation have also been recorded in studies of effects of administration of NSAID to calves after hot-iron disbudding. As shown in Table 2, the heart rate of calves in the hours post-disbudding has been studied, and some papers report reduced heart rate in NSAID-treated calves, as compared to calves receiving only a local anesthetic (52, 61), whereas other studies were not able to show any differences (42, 64, 66). Similarly, for measures of heart rate variability, Stewart et al. (52) showed differences between calves treated with NSAIDs and calves only given a local anesthetic, but Clapp et al. (63) did not find any effects (Table 2). For data on respiration, Heinrich et al. (61) showed a reduction in NSAID-treated animals from 0 to 6 h post disbudding as compared to calves administered only a local anesthetic, whereas (42) did not find any differences (Table 2).

Changes in ocular temperature is another consequence of sympathetic activation and vasoconstriction that has been quantified by infrared thermography in studies of removal of horns in calves (37, 41, 52). However, this also gave unclear results (52). The finding that changes in ocular temperature co-occurred with the changes in heart rate variability, however, may suggest that both are measures of sympathovagal imbalance (44). As listed in Table 2, changes in ocular temperature have been included in studies of effects of NSAIDs on responses to hot-iron disbudding in calves, but no clear findings have been presented.

### Blood parameters

The plasma concentration of cortisol is the most commonly studied indicator of welfare aspects of horn removal in calves. With only a few exceptions [as discussed by (52) and reviewed by (44)], the vast majority of the studies have shown that removal of horns in calves—irrespective of the applied method—leads to a marked, early increase in the plasma concentration of cortisol [e.g., (39, 41, 76). The concentration typically decreases after ~7 h (27, 33, 78)]. Studies involving the administration of a local anesthetic before the procedure show—again almost unanimously—a smaller cortisol peak after the procedure as compared to calves dehorned without any pain relief (41, 79, 80). However, the plasma concentration of cortisol remains at a plateau concurrently with the fading of the effects of the local anesthetic (33). Some studies have reported the area under the curve (AUC) of the cortisol profile, and found that it did not differ between calves, where horns were removed without pain relief and calves given local anesthetics. The initial concentration of cortisol was lower with local anesthetics, but the concentration remained increased for a longer time, perhaps due to the pain experienced when the effect of the local anesthetic waned off [reviewed by (12, 44)].

In studies of NSAID pain relief after disbudding, the majority have reported a reduction in the plasma concentration of cortisol in calves administered NSAID (40–42, 58, 61, 64) (Table 2). However, the reported duration of the effect differs somewhat between the studies.

From human and veterinary patients it is known that burn injuries induce profound inflammation [as reviewed by (72)], and measures of inflammatory markers in blood have been included in studies of removal of horns in calves. Among the examples are plasma concentration of Substance P (41, 64), haptoglobin (41), prostaglandins (PGE_2_) (41), or circulating neutrophiles (51). Measures of inflammatory markers have been included in studies of the effect of NSAID on post-disbudding responses in calves. Allen et al. (41) showed reduced Substance P at 120 h post-disbudding, whereas Stock et al. (64, 66) did not find any differences. One study involved the plasma concentration of haptoglobin, however, no effects of the use of NSAID on calves at disbudding were found (41). As would have been expected, based on the knowledge of the mechanisms of action of NSAIDs, clearer results have been found regarding the plasma concentration of prostaglandins, where (41), and Stock et al. (64, 66) showed significant reductions in calves treated with NSAIDs. From studies in humans and rodents, it is known that NSAIDs may positively affect healing of some tissue types [bone (81), cutaneous wounds (82)], and does not affect others [tendon and ligaments (81)]. At present similar knowledge is not available in calves.

### Production indicators

The main production indicator reported from studies of removal of horns in calves is changes in body weight (quantified as average daily gain, ADG) (41, 43, 64, 65), typically showing lower ADG or tendencies to lower ADG in calves without horns as compared to intact controls. However, long-term effects beyond a few weeks have not been examined.

Table 2 lists the available papers reporting weight gain of the calves in studies comparing calves treated with a local anesthetic and a NSAID vs. only local anesthesia. Weight gain has been quantified as the difference between body weight before disbudding and the body weight after some days, and the studies differed considerably in the chosen interval between weighing (from 24 h to 30 days). No significant effects of the NSAIDs were found. Intake of either milk or concentrate are obviously related to growth and have been addressed in studies of the effects of NSAID on calves post-disbudding (38, 58, 59, 62). Heinrich et al. (38) and Duffield et al. (59) reported tendencies for an increased feed intake in NSAID treated calves (Table 2).

### Indicators of affective states

As reviewed by Mendl et al. (83), the term sentience may cover many different states in humans and animals, of which pain is one highly important negative affective state, especially when concerning a tissue damaging management procedure such as removal of horns. Across humans and animals, the term pain covers an unpleasant sensory and emotional experience associated with actual or potential tissue damage or described in terms of such damage (84). However, quantification of pain in non-verbal individuals is not simple (85) and must be assessed indirectly.

One initial way to gain knowledge about animal pain is by asking caretakers and veterinarians about their experiences with animals undergoing a certain tissue-damaging procedure. Across countries and regions, farmers as well as veterinarians consider removal of horns a painful procedure for calves. In a questionnaire survey, Huxley and Whay (8) asked UK veterinarians about the level of pain experienced by calves during hot-iron disbudding on a scale from 0 to 10, and scored a median of 7 (range 2–10). In a comparable Canadian set-up authored by Hewson et al. (9), the reported mean score was 7.2 (range 6.9–7.5) when no pain relief was given to the hot-iron disbudded calves. More recently, Fajt et al. (10) reported a median score of 6.3 from US veterinarians, and Wikman et al. (11) found a score of 9 when asking Finnish beef producers. Recently, Hambleton and Gibson (3) surveyed UK veterinarians and farmers as part of one of the first studies examining the opinion of stakeholders regarding effects of NSAIDs in terms of post-disbudding pain in calves. Based on their results, the farmers gave a lower pain score for calves treated with a local anesthetic in combination with NSAID as compared to the anesthetic alone, whereas the veterinarians did not.

Behavioral measures are considered to be among the strongest types of indicators of animal pain (44). When calves are disbudded, their immediate response to the hot-iron involves behaviors such as escape attempts, struggling, rearing and falling, all suggested to indicate severe (12, 13) or intense (86) pain. These responses are seen to a much lower extent when the calves have received a local anesthetic.

Among the available studies of behavior after horn removal in calves, the majority have focused on expressions of so-called “pain behavior” (32) or “pain-related behavior” (42), such as ear shaking and head rubbing. In principle, terms such as “pain-related” are not correct [as suggested by (87) and further discussed by (88) and (89) for pigs], because the single behavioral elements may have rather different underlying motivations and thresholds of expression. In calves, these behaviors are shown in the hours after disbudding (76). “Pain-related” behaviors have also been recorded in studies of post-disbudding effects of NSAIDs. The occurrence of behaviors such as head shaking, ear flicking, or head rubbing were significantly reduced after administration of an NSAID (38, 40, 46, 59) (Table 2). The reported duration of the effects differ somewhat between the studies, e.g., from 24 h (40) to 44 h (38), and in all cases the effects were still present at study termination.

At present, no validated pain scales are available for calves. Recently, a multidimensional score for post-disbudding pain in calves were suggested (75) but it has not been validated yet. Braz et al. (90) created a numerical rank scale (0–10) based on the scoring of calf behavior (including observations of head shakes, head rubs, ear flicks, and postural changes) and scored 0–8 for disbudded animals. By use of the scale, they were able to separate sham treated and disbudded calves during all their 15 min observation intervals covering the initial 60 min after chemical disbudding.

In recent years, paradigms originally developed in human and animal model studies of psychology, such as cognitive bias, have received increased scientific attention in studies quantifying the affective states of animals (91). A cognitive bias refers to the systematic pattern of deviation from norm or rationality in judgement, whereby inferences about other individuals and situations may be drawn in an illogical fashion. Today, studies within the animal welfare literature have provided evidence for cognitive bias in animals exposed to housing or management practices, such as chickens exposed to isolation (92). Thus, studies of cognitive bias are not quantifying pain as such but seek to assess the emotional valence of an experience (93). Recently, Neave et al. (94) studied cognitive bias in calves before and after hot-iron disbudding. They found a significantly negative cognitive bias in the response of the calves to an unambiguous stimulus after disbudding (tested at 6 and 22 h post-disbudding), thereby suggesting that hot-iron disbudding induces a state of negative expectations or negative pessimism-like mood in calves.

Another motivational paradigm to study affective states in animals is the use of conditioned place avoidance. Here, the effects of an aversive experience are tested when the animal is no longer in the presence of the stimulus, and the observed responses therefore cannot be explained by e.g., attempts to escape from the aversive situation [as reviewed by (95)]. Wong et al. (96) used this approach to compare responses to various chemical agents used to anesthetize laboratory zebrafish and recently, Ede et al. (97) used a comparable set-up to present evidence to suggest that intra-muscular injections of saline were considered more aversive by calves than for example subcutaneous injections.

In contrast to the studies of negative affective states such as pain, the study of indicators of positive affective states has only recently started. As mentioned previously, play behavior has been suggested as a candidate indicator for positive affective states in juvenile (26).

Despite the recent scientific attention directed toward the use of motivational paradigms such as cognitive bias (91, 93) or conditioned place avoidance (95, 96), none of these or comparable methodologies have yet been applied in studies of the effects of NSAIDs on post-disbudding responses in calves.

## Discussion

In this review, we have sought to include measures of the three major welfare concerns (affective states, basic health, and functioning and naturalness) when candidate measures for these have been available. The majority of available evidence suggest that adding the use of a NSAID to the administration of a local anesthetic is advantageous in terms of animal welfare, and no studies suggest that NSAIDs are a disadvantage to animal welfare. Recently, Winder et al. (50) came to similar conclusions. Below, we discuss how well the different types of indicators and especially the welfare concerns are represented in the available data and the potential consequences of this. In addition we seek to identify areas where research is needed in order to fully understand and quantify the welfare effects of hot-iron disbudding in calves—even after administration of a combination of local anesthetic and NSAID.

### Does lack of horns matter?

As mentioned, removal of horns breaches the integrity of the animals, and can be described as a way of adapting the animals to the production conditions. Hence, even though body integrity was not mentioned as key element of naturalness by Ventura et al. (24), the mere removal of horns might—from a naturalness perspective—be reducing the welfare of the calves. Paradoxically, despite the numerous studies focusing on removal of horns in cattle published during the last decades [as reviewed by (13, 44, 47)] very little is known about the function of horns in domestic cattle [as discussed by (4). Cattle may be naturally polled (98) and recently breeding for polled animals have gained interest due to concerns about animal welfare (1, 99) and might be an economical attractive alternative to dehorning (100). Goonewardene et al. (101) compared the behavior of polled and dehorned cattle and found no difference in their response to handling. Only very few studies have compared the behavior of horned vs. dehorned animals and if so, almost exclusively in beef cattle under conditions differing substantially from the typical, modern dairy production [as reviewed by (4)]. Hence, at present only the procedures of horn removal have been studied, but not the potential welfare consequences for the animals in terms of living with their horns removed at a young age.

Based on an underlying utilitarian ethics, as exemplified by Kupczynski et al. (102), different management procedures—even tissue damaging—can be legal in farm animals, as long as the alternatives are considered to have worse consequences for animals and/or humans (103). Removal of horns in cattle is justified by for example reduced risk of damaging humans, other cattle or even other animal species [as suggested by (12)] as well as facilitating cattle handling [as suggested by (102)]. However, as for the function of horns, negative consequences of having horned animals in modern dairy cattle production are not well-documented and the majority of the available studies were performed years ago under conditions that differed considerably from modern dairy production (104–106). Recently, Irrgang et al. (107) examined effects of space allowance on antagonistic interactions in horned dairy cows waiting for milking, but did not include dehorned animals in the study. Kling-Eveillard et al. (108) interviewed farmers from selected European countries, where the keeping of horned cattle is relatively common, and concluded that managing animals with or without horns is rooted in different views on the farming profession, on the human-animal relationship, and on the practical and daily work with the animals. Hence, even though non-relieved pain during and after removal of horns in calves most often is considered a welfare problem—based on the concern for animal affective states (14)—the removal of the horns as such may also be considered a problem due to other concerns such as naturalness and this is not solved by use of NSAIDs and local anesthetics.

### The comparability and generality of the available studies

This review clearly suggest that the use of NSAIDs in combination with local anesthetics is advantageous. However, despite the relative strength of the conclusion, the available studies are characterized by a low degree of comparability, as they most often have not followed similar, validated protocols and differ considerably in their choice of outcome measures and choices of experimental set-up. In a systematic review of pain management in neonatal pigs during routine management procedures, Dzikamunhenga et al. (87) came to comparable conclusions, and stated that confident decision making is then challenged by the lack of possibility to compare results between studies. Similarly, in their recent systematic review on pain relief in calves after disbudding, (50) recommends consideration of more standardized outcome measurements, especially for pain behaviors as well as adherence to reporting guidelines.

As an example, it is clear from Table 2 and the review of the studies above, that the studies differ considerably in terms of age of the calves or the calf age interval used. Even though the use of a wide range of age groups may increase the external validity of the results, it becomes very difficult to compare studies. Hence, at present evidence is not available to suggest whether calves of certain ages will benefit more or less from the use of NSAIDs in combination with a local anesthetic for hot-iron disbudding. Stafford and Mellor (13) came to a similar conclusion regarding possible interactions between calf age and other aspects of the different horn removal procedures and analgesic protocols. Recent work by Stock et al. (44) underlines this, as they discussed how changes in plasma concentration of cortisol might vary according to the age of calves.

The lack of comparability across the available studies is not limited to the animals included in the studies. As reviewed by Adcock and Tucker (47), even within studies of hot-iron disbudding, the horn removal procedures and equipment is not similar and the choices often not scientifically justified. The procedures are often not described in details, but it cannot be excluded that potential differences in for example hot iron tip dimensions, the heat capacity of the hot iron, duration of the application, or the pressure applied may underlie some of the conflicting results shown in Table 2.

One further challenge for the comparability of the available studies is the lack of a common, validated protocol for behavioral recordings and the lack of justification of the chosen recording and sampling rules. For example, other types of sampling than scan sampling may be advantageous for recording of short lasting behaviors such as head shaking or ear flicking (as for instance done by Sylvester et al. (31) in a study of amputation dehorning or (51) in a study of hot-iron disbudding).

### Are the welfare concerns and different welfare indicators examined in the literature on calf disbudding?

From the review above and the results summarized in Tables 1 and 2 it is evident that the vast majority of the scientific focus has been directed toward the welfare concern of the affective state—mainly focusing on pain quantified by the occurrence of so-called “pain-related” behaviors. In order to be able to incorporate all welfare concerns, and fully understand the consequences of removal of horns in calves, and the potential benefits from using NSAIDs combined with local anesthesia, future studies should involve indicators of other welfare concerns as well. In addition, future studies should take advantage of the recent developments in the quantification of affective states in animals [as done by (94)] and aim to set up trials to identify the types of emotion felt by the animals and especially the emotional valence [as discussed by (95)].

In section Does Lack of Horns Matter? aspects of lack of naturalness as a consequence of calf disbudding were discussed, but as shown in Table 1, only few indicators of naturalness have been examined in studies of the welfare consequences of this procedures. Compared to the concern for naturalness, the welfare concern for basic health and production of animals has received more attention in studies of removal of horns in calves. However, this has mainly been through the inclusion of physiological indicators such as cortisol [e.g., (40, 42, 66)] or heart rate [e.g., (52, 61, 64)], which may not be clearly linked to productivity, and which are also used as indicators of affective state. As can be seen in Table 2, no clear evidence regarding the benefits of the use of NSAIDs combined with local anesthesia in terms of ADG—one very direct measure of productivity—has been presented. It is possible that restricted access to milk in the studies involving recording of bodyweight changes of the calves has limited the overall potential for weight gain—the calves may simply not have been able to gain more weight on their restricted diet [as suggested by (65)]. In order to proper characterize the welfare effects of removal of horns in calves and potential benefits from the use of NSAIDs combined with local anesthesia, future studies should focus on measures of productivity, preferentially in set-ups where the weight gain potential of the calves are not limited by other factors. In a study of chemical disbudding, Vasseur et al. (109) compared responses of high and low fed calves and found that the calves fed the low milk ration responded stronger to the removal of the horns. Hence, it is possible that non-pharmacological interventions may reduce the negative consequences of the tissue damaging procedure for the calves. However, despite this potential, so far, no studies have combined pharmacological and non-pharmacological interventions.

As discussed by Cardoso et al. (110) in a study of Brazilian dairy farmers, this group of stakeholders may trade animal welfare with production goals. Hence, future focus on productivity may include studies of the costs of the use of the medication protocols—direct as well as indirect—as for instance Hötzel and Sneddon (111) showed that Brazilian extensionists were reluctant regarding the use of analgesics for removal of horns based on arguments of increased labor and costs. Based on Canadian stakeholder meetings, however, Ventura et al. (24) suggested that the current North American lack of analgesic use is out of step with values within the dairy industry and in a similar set-up Robbins et al. (112) suggested that the practice of horn removal without pain management is not consistent with normative beliefs. Irrespective of possible regional differences, more knowledge of direct and indirect costs of the use of NSAIDs in combination with local anesthetics will be advantageous.

Among the different types of welfare indicators, clinical indicators may be highly relevant for the concern of health and productivity. Paradoxically, in the days and weeks after removal of horns in calves, very little is known about changes in classical clinical measures, such as for instance healing of the lesions (68). In order to fully understand the welfare effects of hot-iron disbudding in calves and the combined use of local anesthetics and NSAIDs, future studies should include clinical aspects such as healing. Often, clinical measures are considered relevant also for the other welfare concerns, and inclusion of this type of indicators would, thus, allow conclusions across welfare concerns.

To the best of our knowledge, no horn removal studies so far have justified their choice of outcome measures based on the different welfare concerns or explicitly included indicators from all categories listed in this review. The latter should not be an aim in itself, but may be one way to provide evidence of relevance for the different welfare concerns. Weary et al. (113) discussed the importance of such knowledge for the possibility to implement science-based recommendations, using the paradox of the clear science-based welfare benefits from use of enriched cages for layers, and the concomitant lack of implementation of this type of housing as an example. Among the recent studies of removal of horns in calves, there seem to be an increased focus on the inclusion of several indicators, often reflecting more than one welfare concern. One such example is Winder et al. (32), who studied chemical disbudding and included measures from four of the five abovementioned categories of welfare indicators (MNT, play, heart rate, respiration, milk intake, time spent lying, and latency to approach a human).

### Are calves experiencing pain despite combined use of analgesics and NSAIDs?

The above review shows almost unidirectional positive effects of the use of NSAIDs combined with local anesthetics to calves after hot-iron disbudding (Table 2). However, in humans management of pain after burn injury is considered an unresolved and complicated clinical issue (72, 114–116). Importantly, burn pain is not like surgical pain, as it is highly variable, and may increase over time. Summer et al. (72), and more recently (116), reviewed how unrelieved pain from human burn injuries is believed to contribute to long-term sensory problems, including chronic pain, paresthesia, and dysesthesia as well as debilitating psychological conditions. There are numerous studies of the healing of and pain from burn injuries in humans and animal models [as reviewed by (115)], but the lesions inflicted by hot-iron disbudding in calves does not fall in either of these types of studies, and generalization between them may thus be difficult. In the typical animal model study of thermally induced injury, the animals (rodents or pigs) are exposed to damage of a much lower degree than after hot-iron disbudding (such as lowering a paw into 50°C water) (117, 118). Broadly, the human studies are either—for ethical reasons—model studies of rather limited injuries [e.g., (119)], or clinical studies of burn patients after accidents, typically involving major burn injuries covering a much larger proportion of the body than after hot-iron disbudding [e.g., (120, 121)]. Nevertheless, there has been a methodological flow toward cattle studies including measures such as changes in nociceptive thresholds (25, 75, 122) or electroencephalographic responses (36). The use of methodology, which—at least to some extent—is overlapping may, now and in the future, facilitate comparison between studies of hot-iron disbudding in calves and studies from other species and, thus, improve the understanding of the welfare effects of horn removal.

From the above review it is evident that the majority of the available studies have focused on effects observed within hours (52, 58, 59) or a few days (38, 41, 62) after the procedure. Further knowledge about effects of hot-iron disbudding in terms of pain might be obtained by focusing on the available knowledge about burn lesions—such as the different types of pain and the different phases of healing. Summer et al. (72) reviewed three distinct phases after human burn injury: the acute phase, the healing phase and the rehabilitation phase. These may not have comparable consequences in terms of animal welfare and may not be recognizable by use of similar pain indicators when studying animals. From a clinical perspective, the acute phase of burn injury in humans often focus on limiting the risk of shock and stabilization of the patient, but for calves after horn removal, where the injury typically is small compared to human burn patients, this part may not be central. Concerning the acute pain, some human patients report immediate pain, whereas others are not experiencing the pain until after some hours. The healing phase can last weeks or longer, and the rehabilitation phase lasts even longer. In a review of human burn pain, Latarjet and Choinere (123) stated that pain is present on a daily basis in the weeks after a burn injury. Whether this is the case for calves after hot-iron disbudding is not known and should be studied further in order to fully understand the welfare effects of the tissue damaging management procedure as well as potential benefits from the use of NSAIDs combined with a local anesthetic. Here, it is important to acknowledge that the developing pain is not constant and thus, indicators might need to adapt to the underlying process.

In human burn patients, the healing and rehabilitation phases of the injuries are characterized by pruritus gradually replacing pain (60–87% of patients report persistent or intermittent itch) (72). Itching and tingling sensations often accompany the healing process and may sometimes be experienced as equal in discomfort to the pain itself (123), and hence be of high relevance for the welfare of the patient—human or animal. Across humans and animals the study of itching is increasing (124, 125), but at present only very little is known about itching in cattle and how this animal species experience itching and express it behaviorally or physiologically. In the studies reviewed above, few have included self-grooming (46, 58, 59), but most probably too early in the healing phase to be able to document increased itching. Stafford and Mellor (13) discussed the possible change from pain to other discomfortable experiences such as irritation or itching. In these years, cow brushes receive scientific attention [e.g., (126, 127)] and recently Zobel et al. (128) reported that calves do use automated brushes. Comparable equipment may be used to seek information about potential consequences of hot-iron disbudding on pruritus in calves. Whether the use of NSAIDs in combination with local anesthetics for post-disbudding analgesia affects the occurrence of pruritus in the weeks after the procedure is unknown.

Across the studies reviewed above, none has focused on different types of pain being present at the same developmental stage of healing of the injuries. In humans, irrespective of the three phases of burn lesion healing, the pain experienced by the patients is categorized into procedural pain (when hospital staff or the like handles wounds), background pain and breakthrough pain (71, 72). Procedural pain is described by patients as pain of an intense burning and stinging quality, lasting for minutes or even hours after for example dressing changes. In calves, the disbudding injuries are typically not handled as such, but the affected skin area might be subject to tactile stimulation from other calves or from for instance the drinking of milk. Just lowering the head, and thereby increasing the pressure associated with venous distention of the inflamed tissue, may also be somehow comparable to procedures experienced by hospitalized human patients. So far, none of the available studies of responses to removal of horns in calves has included quantification of social contact, cross-sucking or behavior during milk drinking. Housing cows and calves together after hot-iron disbudding could be one model to examine whether disbudding affects the natural behavior of calves with or without the use of NSAIDs and thereby obtain knowledge relevant for the welfare concern for naturalness. To the best of our knowledge, no such studies have been done yet.

The second type of pain is background pain (72). Background pain is reported by patients to be of prolonged duration, relative constant nature, and of mild to moderate intensity. In addition, this type of pain has been described as a continuous burning or throbbing pain that is present even when the patient is relatively immobile. In the acute phase, this pain can be from none to severe, and may be worsened by exposing the wound to air. None of the studies reviewed above has focused directly on background pain, but occurrence of some of the behavioral variables already studied—such as lying, inert lying, feeding or self-grooming (Table 2)—may be potential candidates for documentation of such pain. However, as discussed above for affective states in general, studies of this type of pain will probably warrant the use of continuous behavioral recordings for considerable periods of time.

The third type of burn injury pain is breakthrough pain (72), a transient worsening of the pain, most frequently associated with movement—typically termed end-of-dose breakthrough pain when the waning of the effects of analgesics coincide with reoccurrence of pain. Without using the term as such, some of the above reviewed studies of the effects of combined use of NSAIDs and local anesthetics have focused on this type of pain [e.g., (41, 52)] and found that the addition of NSAID has a normalizing effect of the changes in plasma concentration of cortisol.

### For how long after hot-iron disbudding may the calves experience pain?

Across all types of horn removal included in this review, the duration of the pain after the procedure is not yet known. Recently, Knierim et al. (4), Adcock and Tucker (47), and Winder et al. (50) came to the same conclusion. Based on the combination of behavioral and physiological indicators, Stafford and Mellor (13) suggested that the pain might continue for up to 72 h. However, this question is highly central for the potential benefits of the use of NSAIDs combined with local anesthetics and needs to be examined further. The effect of a single injection of NSAID varies considerably in time; the longest acting products at the Danish market are Meloxicam with a half-life of ~24 h (129) and Carprofen with an even longer half-life (130).

In human burn patients, chronic pain or sensation changes are considered a significant problem after burn recovery (131). Typically, the healed skin presents as hyper- or hyposensitive areas which may develop into chronic neuropathic-like pain (123). Only rather recently, measures of changes in nociceptive sensitivity quantified by algometers (38, 41, 64, 66) or von Frey filaments (25) have been included in studies of removal of horns in calves. From these studies, changes in sensitivity have been documented up to 75 h (25) or even 105 days (122) thereby suggesting that calves show acute as well as persistent sensitization after hot-iron disbudding. These results call for further studies of the consequences of hot-iron disbudding in terms of changes in nociceptive thresholds.

### How can this knowledge be implemented?

Recently, Hambleton and Gibson (3) reported results from a questionnaire survey among UK stakeholders, stating that 56% of the responding veterinarians and 22% of farmers were positive toward the use of NSAIDs in combination with local anesthesia. Forty-nine percent of the veterinarians stated that they believed that the use of NSAIDs should be mandatory. Even though these new results are not directly comparable to older surveys, it is likely that the proportion of veterinarians already using NSAIDs has increased considerably during the last decade [(8) reported 2%]. Interestingly, farmers and veterinarians differed in their reporting of experiences with the effects of NSAIDs, as farmers reported a positive effect whereas veterinarians did not. The reasons for this difference is unknown. It is possible that the available results reviewed above (of which some are more than 10 years old) do not find their way into cattle practice. Also, farmers have more practical experience with the long term effects of NSAIDs as they spend time with the calves in the hours and days after horn removal, whereas veterinarians typically only observe the animals during the procedure.

At present, little is known as to whether a single dosage of NSAIDs in combination with a local anesthetic is sufficient. Of course, this will also depend on the choice of medication protocol as the pharmacokinetics differ considerably. Orally administered Meloxicam, for instance, has been shown to have a rather slow elimination phase with a half-life of ~24 h (129, 132). Accordingly, the physiological and behavioral effects of a single preoperative dose of the longer-acting NSAID Meloxicam have been shown to persist for more than 1 day after hot-iron disbudding (38, 41). On the other hand, Flunixin meglumin given intravenously has a half-life of 3–8 h, and additional administration will be needed to obtain similar effects (42, 133). Further studies are needed in order to clarify the optimal timing (e.g., possible benefits from preventive pain management), dosage, drug and type of administration of NSAIDs combined with local anesthetics in order to improve the post-disbudding welfare of calves. The recent results by Ede et al. (97) quantifying the degree of aversion related to different types of injections, suggest that possible welfare consequences of the administration of the NSAID as such—at least when injections are used—should be included in this work.

## Conclusion

Based on three major welfare concerns and different types of animal-based welfare indicators, we have reviewed welfare aspects of hot-iron disbudding in calves focusing on available evidence for effects of the use of NSAIDs combined with a local anesthetic vs. performing the procedure after administration of a local anesthetic only. The majority of available evidence suggest that the use of NSAIDs combined with a local anesthetic is advantageous in terms of animal welfare, and no studies suggest that NSAIDs are a disadvantage to animal welfare. However, irrespective of the welfare concern, the use of NSAIDs does not eliminate the welfare challenge from hot-iron disbudding. Further research is needed in order to fully understand the effects of NSAIDs on calf welfare, including knowledge of the duration of healing and the possibility of long-term pain. At present, this lack of knowledge challenges the precise formulation of the adequate pain management—in terms of medication protocol, duration, dosage, and type of administration.

## Author contributions

All authors listed have made a substantial, direct, and intellectual contribution to the work, and approved it for publication.

### Conflict of interest statement

The authors declare that the research was conducted in the absence of any commercial or financial relationships that could be construed as a potential conflict of interest.
